# Poor consideration of tissue loading in randomised trials of MSC interventions for tendon pathology: A systematic review using the TIDieR framework

**DOI:** 10.1002/jeo2.70388

**Published:** 2025-07-27

**Authors:** Ben Dyck, Chris Clifford, Gordon J. Hendry, Graeme P. Hopper, David F. Hamilton

**Affiliations:** ^1^ Research Centre for Health Glasgow Caledonian University Glasgow Scotland UK; ^2^ Department of Physiotherapy NHS Lanarkshire University Hospitals Scotland UK; ^3^ Department of Trauma & Orthopaedics NHS Lanarkshire University Hospitals Scotland UK

**Keywords:** clinical trial, mesenchymal stem cell, rehabilitation, tendon

## Abstract

**Purpose:**

Mesenchymal Stem Cell (MSC) interventions are a new frontier in the clinical management of tendon injury. In terms of tissue repair and regeneration, both tendon cells and stem cells are mechanotransductive, i.e. they require mechanical stimulus, it therefore follows that well‐structured post‐intervention rehabilitation is needed to support MSC interventions and should be well considered in MSC clinical trials. This review evaluates the completeness of reporting of rehabilitation following MSC interventions for tendon pathology in clinical trials.

**Methods:**

A systematic review of randomised controlled trials was conducted in line with the Preferred Reporting Items for Systematic Reviews and Meta‐Analyses (PRISMA) guidelines and using the Template for Intervention Description and Replication (TIDieR) framework. We applied a PICO framework to inform our search strategy to find clinical trials that used either bone marrow or adipose‐derived MSCs as an intervention on human tendons. Electronic searches were conducted in Medline, PubMed, CINAHL and SPORTDiscus, from inception to May 2024. MeSH terms and Boolean operators were employed, with English language the only filter. Data was extracted to complete the TIDieR checklist separately by three researchers and cross checked by a third to ensure consistency. The Cochrane risk of bias tool 2 was employed to review trial internal validity.

**Results:**

The search returned 142 articles. Following removal of duplicates, 118 papers were evaluated against the inclusion criteria. Eight RCTs were included, comprising five in rotator cuff pathology and individual trials in Achilles, gluteal and patellar tendinopathies. Various MSC preparations were utilised and reported, however the accompanying rehabilitation framework was poorly described with a mean TIDieR score of 2.38 ± 2.56 points (of a maximum of 12). The maximum score was 6/12 for a single trial, while 3 scored 0/12. There was large variability in rehabilitation reporting, however ‘why’, and ‘where’ domains were reported in only 1 study, with ‘tailoring’, ‘modifications’, ‘adherence’ and ‘fidelity’ TIDieR domains not reported in any trial. The included studies demonstrate a high risk of bias. Concerns regarding participant randomisation, participant blinding and group allocation were common across the included studies.

**Conclusion:**

Current randomised controlled trials demonstrate a poor standard of reporting of physical rehabilitation following MSC interventions for tendon pathologies, and highlight the lack of consideration given to post intervention loading. More comprehensive trials that fully incorporate post‐MSC loading parameters are required to better understand MSC efficacy in tendon repair.

**Level of Evidence:**

Level II, therapeutic studies.

**Abbreviations**
BMAC: bone marrow aspirate concentrate
BM‐MSCs: bone marrow‐derived mesenchymal stem cell
MSC: mesenchymal stem cell
PRISMA‐ScR: Preferred Reporting Items for Systematic Reviews and Meta‐Analyses extension for Scoping Reviews
RCT: randomised controlled trial
ROB‐2: Cochrane Risk of Bias tool 2
TIDieR: template for intervention description and replication

## BACKGROUND

Tendons are some of the most commonly injured structures in the body; damage to which represents a major societal burden in terms of impacted quality of life, work absenteeism and treatment cost [20, 23, 28, 34]. Damaged tendons heal slowly and typically do not regain the structural integrity and mechanical strength of the pre‐injured tendon [37]. Injury management tends to relate to the severity of tissue disruption, centred around physiotherapy rehabilitation and surgical intervention [1, 6, 32], however the effectiveness of these management strategies is limited [14]. Recent advances in stem cell therapies have challenged the traditional approach to tendon injury management with numerous authors investigating whether biological therapies can supplant pre‐existing strategies regardless of injury severity [6, 27]. These new therapies aim to combat historically poor outcomes with tendon rehabilitation by regenerating the injured tendon tissue [6, 22, 37].

Acute tendon injuries create an inflammatory response leading to collagen formation and remodelling, a process that may take years to fully mature [11]. The primary inhibition and limitation to this intrinsic repair is a lack of mechanical stimulus applied to the tendon during the repair phase, as a foundational component of tendon strengthening occurs in response to mechanical loading [11, 35, 37]. This requirement of mechanical loading is essential regardless of the type of tendon injury. At a cellular level, mechanosensitive tendon tissue responds and adapts to the load demands placed upon it by altering collagen synthesis and tissue remodelling. Indeed, a base level of physiologic load is required to maintain tendon homoeostasis in healthy tendons [11]. Following tendon injury this need for mechanical loading is increased further, as tendon cell deformation and subsequent adaptation is load‐dependent [2]. This load‐dependent tissue adaptation is the cornerstone of tendon injury rehabilitation, in both acute management and following surgery, where a protocol around specific mechanical loading can improve repair as well as an individual's pain and functional outcomes by leveraging the response of tendon tissue to load [2, 3, 14].

Stem cell augmentation aims to support current approaches to tendon repair, increasing the regenerative capacity of tendon tissue by supplementing the body's natural regenerative capability [16, 33]. Mesenchymal stem cells (MSCs) can differentiate into different types of cells depending on the stimulus they are exposed to [9]. While MSCs may be obtained from a variety of sources, trials examining their role in tendon repair tend to source MSCs through either bone marrow aspirate concentrate (BMAC) or local adipose tissue (adipose stem cells; ASC) which are then processed via stromal vascular fraction [33]. MSCs possess the same capacity for differentiation regardless of source [33], although the concentration of available MSCs is up to 6.5 times greater in adipose tissue compared to bone marrow; which, combined with a significantly less invasive harvesting process, has led to a preference for ASC in human trials [1]. Within the context of tendon repair, MSCs respond to mechanical stimulus just as native tendon cells do, enhancing the potential for MSC augmented tendon repair [35].

Biological therapies have become increasingly popular within sports medicine, and biological treatments are widely used despite a lack of high‐quality evidence as to efficacy [5, 32]. The mechanotransductive nature of both tendon tissue and MSCs necessitates a mechanical stimulus to potentiate both native tendon and MSC proliferation and strengthening [2, 11, 22, 35, 37]. However, it is unclear whether such a structured approach to tendon rehabilitation following MSC injections is being properly implemented in clinical trials. Recent reviews examining MSC interventions for tendon disorders [19, 27, 32] have not included reports of physical rehabilitation or tissue loading protocols following intervention. As such, the aim of this work was to review the reporting and quality of the rehabilitation protocols that accompany randomised trials of MSC interventions to tendon injuries.

## METHODS

A systematic scoping review of randomised controlled trials was undertaken in line with the Preferred Reporting Items for Systematic Reviews and Meta‐Analyses extension for scoping reviews (PRISMA‐ScR) guidelines [30]. Our pre‐registered review protocol is available via the open science framework https://osf.io/b6ptu which includes our study design framework and search strategy.

### Information sources and search strategy

We applied the PICO criteria to inform our search strategy which aimed to identify randomised controlled trials that used either bone marrow or adipose‐derived MSCs as an intervention to investigate their effects on tendon disorders in humans (Supporting Information: Data S1). The specific target of our review is the accompanying rehabilitation frameworks; however, the underlying search is of the trials of the MSC interventions with a specific review of the reporting of the rehabilitation and loading parameters that these trials contained.

The search strategy was devised in conjunction with a specialist librarian and an electronic search of the following databases was conducted from inception to 12 May 2024 in Medline, PubMed, CINAHL and SPORTDiscus. Boolean operators were employed in the searches as detailed in Supporting Information: Data S1. We applied an English language restriction but no other filters to the search. Manual searches of Google Scholar and citation searching of included manuscripts were also completed.

### Eligibility criteria and study selection

Eligibility criteria are defined by the review PICO. All randomised controlled trials describing a bone marrow or adipose‐derived MSC intervention in humans with tendon injuries were eligible for inclusion. The primary outcome of this review was the reporting of the trial rehabilitation and tendon loading parameters. Where relevant (and if specifically directed in the included trial reports) previously published trial protocols were used as reference material to obtain detail as to these parameters. Exclusion criteria included animal trials, alternative biological agent use (i.e. not bone marrow or adipose‐derived MSCs), non‐randomised trials and trials investigating non‐tendinous structures.

A three‐part screening strategy was employed to identify relevant articles. One investigator carried out the searches and screened by title. Abstracts were reviewed independently by two investigators and consensus reached through discussion for full text inclusion. In the event of disagreement, or doubt, manuscripts were included for full text review. Full texts were reviewed by the same two reviewers independently. In the event of unresolvable differing opinions as to final inclusion, an arbitrator was available for consultation.

### Data extraction

Data were extracted using a bespoke Excel database by two reviewers with agreement by consensus. A third reviewer was available to resolve any disputed issues by simple majority. Data sought included the year of publication, geographic location where the trial was conducted, the injury type (body region and structure), the trial sample size, demographics and group allocation, a description of the trial intervention, the outcome measures employed, and the trial follow‐up period.

### TIDieR reporting quality assessment and risk of bias

Data on quality of reporting of the rehabilitation interventions were separately extracted using the TIDieR checklist [12] by three reviewers with pooling of results performed by an independent arbitrator. The TIDieR checklist consists of 12 items that cover the who, what, where, when, how and why aspects of the intervention under consideration. Specifically questions relate to the name of the intervention; the rationale, theory or goal; the materials used in delivering the intervention; the procedures, activities and processes; who provided the intervention; the mechanism or mode of delivery; the location or setting; the number of sessions; whether the intervention was tailored or personalised; if any modifications were made; if adherence was assessed, and the fidelity achieved (extent to which the intervention was delivered as planned). We scored the selected studies by allocating one point for each parameter adequately described with a maximum possible score of 12. No points were awarded to questions where if the information was absent or insufficient for replication. Information was accepted if it was available within the published paper, or if separately provided in publicly available trial protocols or additional documentation, but only in cases where the published trial report specifically referenced such documents.

Risk of bias within the included randomised trials was assessed using the Cochrane Risk of Bias (RoB2) tool [29]. This tool assesses five major domains, identifying bias that (1) arises from the randomisation process, (2) arises due to deviations from the intended interventions, (3) arises due to missing outcome data, (4) arises due to measurement of the outcome, or (5) arises due to selection of data for the reported result. Each domain is labelled as either low, high, or some concern, with a flow provided by the RoB2 guidance sheet outlining how to synthesise the results of each domain.

## RESULTS

**Figure 1:**
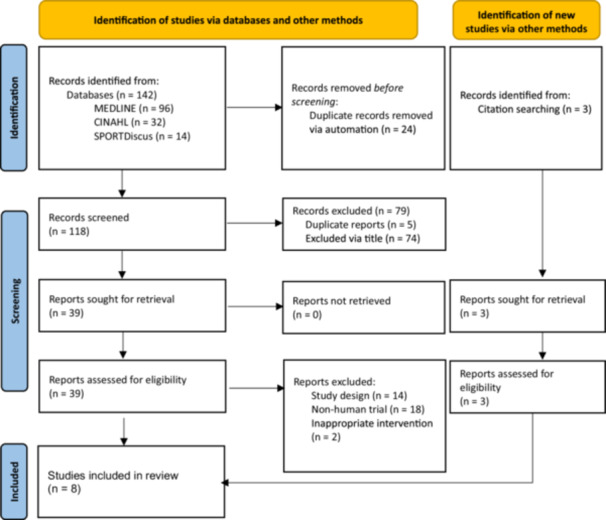
PRISMA flowchart highlighting the study selection process of cell therapy randomised controlled trials for tendinopathies.

The database search returned 142 articles. Following removal of duplicates, 118 papers were evaluated against the inclusion criteria. An additional three studies were identified through reference searching. As such 121 articles were screened and, of these, 42 papers were eligible for full‐text review. Of the 42, eight trials met the eligibility criteria and were included in the final review. Full details are displayed in the PRISMA flowchart (Figure 1).

### Study characteristics

The included RCTs were small trials (*n* = 13–82 participants) that employed various stem cell preparations derived from bone marrow (*n* = 4) or adipose (*n* = 4) cells, which were injected as a treatment (*n* = 5) or as part of a surgical procedure (*n* = 3). Details of the included trials are provided in Table 1. Five trials evaluated interventions for rotator cuff pathology [7, 8, 13, 15, 24], and a single, separate, study assessed patellar [25], gluteal [26] and Achilles [31] tendinopathies. Four studies [8, 13, 26, 31] report an outcome difference in favour of the intervention in some parameters evaluated (at least in the short term) while the other four found no between‐group difference in study outcomes [7, 15, 24, 25].

**Table 1:** included trials demographics, interventions, rehabilitation and outcome measures in cell therapy randomised controlled trials for tendinopathies.

Author (year) location	Injury type	Sample size	Sample demographics	Trial interventions	Trial rehabilitation	Outcome measures	Follow‐up period
Chun [7] (2022)	Partial thickness supraspinatus tendon tear	**Total: *n* = 23**	**Intervention**	**Intervention**	Gentle ROM exercises 3x day	Pain VAS, ASES	6 weeks, 3, 6, 12, 24 months
Korea		Intervention n = 7	Age, 61.0 ± 7.8	ASC in fibrin glue			
		Control n = 8	Female *n* = 4 (57%)	**Control**			
		Active control *n* = 8	**Control**	Saline			
			Age, 54.1 ± 9.4	**Active control**			
			Female *n* = 2 (25%)	Saline in fibrin glue			
			**Active control**				
			Age, 50.4 ± 4.6				
			Female *n* = 4 (50%)				
Cole [8] (2023)	Partial thickness supraspinatus tendon tear	**Total *n* = 82**	**Intervention**	**Intervention**	Standardised protocol:	ASES, SANE, SF‐12,	6, 12, 24 months
USA		Intervention *n* = 42	Age, 56.1 ± 10.1	Arthroscopic rotator cuff repair + BMAC	4–6 weeks in sling gentle mobility	VR‐12, ROM	
					Week 4: PT‐led passive ROM		
		Control *n *= 40	Female *n* = 21 (46%)		Week 8: active‐assisted ROM + isometrics		
			**Control**	**Control**	Week 10+: strengthening + active ROM as tolerated		
			Age, 55.3 ± 9.6	Arthroscopic rotator cuff repair			
			Female *n* = 13 (29%)				
Hurd [13] (2020)	Partial thickness supraspinatus tendon tear	**Total *n* = 16**	**Intervention**	**Intervention**	Advice to avoid strenuous overhead movement and general shoulder activity for 48 hours, then increase activity as tolerated	ASES, RAND‐36, VAS	3, 6, 9, 12, 24, 32, 40, 52 weeks
		Intervention *n* = 11 Control *n* = 5	Age, 62.3 ± 9.6	ASC injection			
USA			Female *n* = 3 (27%)	**Control**			
			**Control**	Corticosteroid injection			
			Age, 57.3 ± 6.3				
			Female *n* = 0 (0%)				
Lamas [15] (2019)	Full thickness rotator cuff tear	**Total *n* = 13**	**Intervention**	**Intervention**	Post‐operative immobilisation in shoulder sling	Constant score, Pain VAS	12 months
		Intervention *n* = 8	57.8 ± 6.5	Rotator cuff repair with collagen membrane scaffold + BM‐ MSCs			
Spain		Control n = 5	Female *n*‐2 (25%)				
			**Control**				
			61.8 ± 3.8	**Control**			
			Female *n* = 3 (60%)	Rotator cuff repair with collagen membrane scaffold			
Randelli [24] (2022)	Full thickness posterosuperior rotator cuff tear	**Total *n* = 46**	**Intervention**	**Intervention**	Standardised protocol:	CMS, VAS, ASES, SST, isometric testing, MRI	3, 6, 12, 18, 24 months
Italy		Intervention *n* = 23	Age, 58.4 ± 7.8	Arthroscopic rotator cuff repair + ASC	28 Days in sling with self‐assisted ROM exercise and elbow + shoulder mobilisation		
		Control *n* = 23	Female *n* = 12 (52%)				
			**Control**	**Control**	Day 29+: Physiotherapist‐assisted rehabilitation for ROM		
			Age, 59.4 ± 6.3	Arthroscopic rotator cuff repair	Month 3+: Physiotherapist‐assisted rehabilitation for strength		
			Female *n* = 15 (65%)				
Rodas [25] (2021)	Patellar tendinopathy	**Total *n* = 20**	**Intervention**	**Intervention**	Standardised protocol:	VAS, VISA‐P, manual dynamometry	3, 8 weeks; 3, 6 months
Spain		Intervention *n* = 10	Age, 35.8 ± 10.0	BM‐MSC injection	• Day 3–14: Isometric range‐controlled quadricep strengthening		
		Control *n* = 10	Female *n* = 0 (0%)				
			**Control**	**Control**			
			Age, 32.0 ± 9.5	PRP injection	• Week 2 or VAS < 4: Concentric range‐controlled quadriceps strengthening, progressing range and load		
			Female *n *= 0 (0%)				
					• Week 4 or VAS < 2: Eccentric range‐controlled compound strength exercises, unilateral strength, walking, running, jumping		
Rosario [26] (2021)	Gluteal tendinopathy	**Total *n* = 40**	**Intervention**	**Intervention**	Unspecified physical therapy protocol + lifestyle change guidance	VAS, Lequesne, EQ‐5D	1, 3, 6 months
		Intervention *n* = 15	Age, 46.1 ± 15.2	BMAC injection			
Brazil		Control *n* = 25	Female *n* = 5 (33%)	**Control**			
			**Control**	Corticosteroid injection			
			Age, 53.2 ± 12.0				
			Female *n* = 15 (60%)				
Usuelli [31] (2018)	Achilles tendinopathy	**Total *n* = 44**	**Intervention**	**Intervention**	None prescribed, participants allowed to gradually resume their normal life and sporting activities	VAS, VISA‐A, AOFAS, SF‐36, MIR, US	15, 30, 60, 130, 180 days
		Intervention *n* = 21	Age, 47.3 ± 3.8	ASC injection			
Italy		(28 tendons)	Female *n* = 7 (33%)	**Control**			
			**Control**	PRP injection			
		Control *n* = 23	Age, 46.6 ± 6.2				
		(28 tendons)	Female *n* = 15 (65%)				

Abbreviations: AOFAS, American Orthopaedic Foot and Ankle Society; ASC, adipose‐derived stem cell; ASES, American Shoulder and Elbow Surgeons; BMAC, bone marrow aspirate concentrate; BM‐MSC, autologous bone marrow‐derived mesenchymal stem cell; CMS, Constant‐Murley Score; EQ‐5D, EuroQol‐5D; MRI, magnetic resonance imaging; MSC, mesenchymal stem cell; PRP, platelet‐rich plasma; ROM, range of motion; SANE, single assessment numeric evaluation; SF‐12, 12‐item short form health survey; SF‐36, 36‐item short form health survey; US, ultrasound; VAS, Visual Analogue Scale; VISA‐A, Victorian Institute of Sport Assessment – Achilles; VISA‐P, Victorian Institute of Sport Assessment – Pain; VR‐12, veterans RAND 12‐item health survey.

### Narrative synthesis

Chun et al. [7] preformed a three‐arm trial contrasting an injection of adipose‐derived stem cells (ASC) embedded within a fibrin glue scaffold with saline embedded within the fibrin glue, and with saline alone for partial thickness rotator cuff tears. No between group differences were seen across the outcome measures employed (*p* > 0.05). Cole et al. [8] compared arthroscopic supraspinatus tendon repair with bone marrow aspirate concentrate (BMAC) compared to arthroscopic repair alone. No between‐group differences in PROM scores (*p* > 0.05) were reported at any time point. A protocol assessing functional strength was abandoned at the 1‐year mark due to the COVID‐19 pandemic, however the authors reported internal rotation at 90° of abduction was significantly improved compared to the control group (*p* < 0.05). Hurd et al. [13] compared the effect of adipose‐derived 'regenerative cells' containing an unspecified quantity of ASCs versus corticosteroid injection for partial thickness rotator cuff tears. The authors report statistically significant between‐group differences in American Shoulder and Elbow Surgeons scores at follow‐up week 24 and 52 (*p* < 0.05), but not for VAS or RAND Short Form‐36 scores at any time point. Lamas et al. [15] contrasted rotator cuff repair with a type‐1 collagen scaffold embedded with autologous bone marrow derived mesenchymal stem cell (BM‐MSCs) and repair with collagen membrane alone. The trial was curtailed early due to adverse outcomes and tendon re‐rupture in both trial arms. With only 13 participants randomised, comparative outcomes were not able to be meaningfully assessed. Randelli et al. [24] performed arthroscopic rotator cuff repair augmented with ASCs compared to arthroscopic repair alone. A statistically significant difference in Constant‐Murley Score favouring the treatment group (*p* = 0.05) was seen at 6‐months, but the difference lost over follow‐up. No other difference in a raft of PROMs was seen at any follow‐up point. No difference was seen in post‐operative MRI review of tendon integrity (*p* > 0.05).

Rodas et al. [25] examined a BM‐MSC injection compared to a PRP control in individuals with patellar tendinopathy. MRI and US imaging at 6 months indicated a significant improvement in tendon structure (*p* < 0.05) in the intervention group, however no between‐group differences were seen in PROMS or strength measures (*p* > 0.05). Rosario et al. [26] compared BMAC injections to a corticosteroid control in participants with gluteal tendinopathy. The authors report significant between‐group improvements in Visual Analogue Scale (VAS) and Lequesne functional index scores favouring the BMAC intervention (*p* < 0.05) but no between‐group differences in EuroQol‐5D scores (*p* = 0.59). Usuelli et al. [36] administered ASCs injections in comparison to PRP in individuals with Achilles tendinopathy. The authors found improved PROM scores in the ASC group within the first 30‐days (*p* < 0.05), but no subsequent differences from day 60 onwards. No difference was seen in magnetic resonance imaging (MRI) or ultrasound (US) imaging at 6‐months (*p* > 0.05).

### Quality of rehabilitation intervention reporting

**Figure 2:**
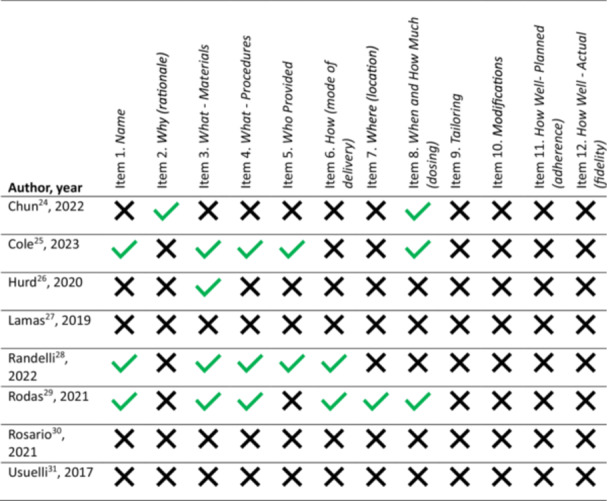
Adherence to TIDieR checklist for included cell therapy randomised controlled trials for tendinopathies.

The rehabilitation frameworks and loading parameters employed in the RCTs were generally poorly described, with a mean TIDieR score of 2.38 ± 2.56 points (of a maximum of 12). The maximum score was 6/12 for a single trial, while 3 RCTs scored 0/12 (Figure 2).

**Figure 3:**
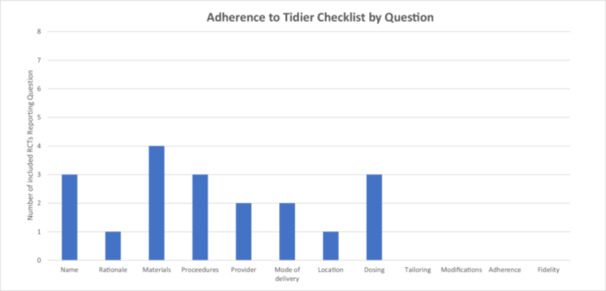
Adherence to TIDieR checklist by individual question for included cell therapy randomised controlled trials for tendinopathies.

There was variability in frequency of reporting of the individual TIDieR checklist items (Figure 3). No category was well reported across the included RCTs. The ‘materials’ domain was most frequently reported with 4/8 studies including some information as to this. Details as to the procedures followed and dosing of the rehabilitation/loading intervention was reported in 3/8 RCTs. Details as to rehabilitation/loading ‘rationale’, and ‘location’ domains were reported in only 1/8 studies, while ‘tailoring’, ‘modifications’, ‘adherence’ and ‘fidelity’ TIDieR domains were not reported in any trial.

The included RCTs demonstrate a high risk of bias, with 6/8 judged to be at high overall risk. Concerns regarding participant randomisation, participant blinding and group allocation were common across the included studies, with a high risk of bias in outcome measurement driving the overall score category (Table 2).

**Table 2:** Risk of bias analysis for included cell therapy randomised controlled trials for tendinopathies.

Lead author (year)	Chun [7] (2022)	Cole [8] (2023)	Hurd [13] (2020)	Lamas [15] (2019)	Randelli [24] (2022)	Rodas [25] (2021)	Rosario [26] (2021)	Usuelli [31] (2018)	
Outcome	Pain VAS, ASES	ASES, SANE, SF‐12, VR‐12, ROM	Primary: Adverse effects	Primary: Constant score, MRI	Primary: CMS	VAS, VISA‐P, manual dynamometry	VAS, Lequesne, EuroQol‐5D	Primary: VAS, VISA‐A, AOFAS, SF‐36	
			Secondary: ASES, RAND‐36, VAS, MRI scan of tear size	Secondary: VAS	Secondary: VAS, SST, ASES, isometric testing			Secondary: MRI, US	
Intervention vs control groups	*Intervention:* ASC in fibrin glue	*Intervention:* Arthroscopic rotator cuff repair + BMAC	*Intervention:* ASC	*Intervention: Rotator cuff repair with BM‐*MSC + collagen membrane	*Intervention:* Arthroscopic rotator cuff repair + ASC	*Intervention:* BM‐MSC	*Intervention:* BMAC	*Intervention:* ASC	
	*Active control:* Saline in fibrin glue	*Control:* Arthroscopic rotator cuff repair	*Control:* Corticosteroid	*Control:* Rotator cuff repair with Saline + Collagen membrane	*Control:* Arthroscopic rotator cuff repair	*Control:* PRP	*Control:* Corticosteroid	*Control:* PRP	
	*Control:* Saline								
	Risk of bias							
Bias arising from the randomisation process	High	Low	Some concerns	Low	Low	Low	High	Some concerns	
Bias due to deviations from the intended outcome	High	Low	Low	Low	Some concerns	Low	High	Low	
Bias due to missing outcome data	Low	Low	Low	Low	Low	Low	Low	Low	
Bias in measurement of the outcome	*Intervention:* low	*Intervention:* low	*Intervention:* high	*Intervention:* high	*Intervention:* high	*Intervention:* low	*Intervention:* high	*Intervention:* high	
	*Active control:* low	*Control:* low	*Control:* high	*Control:* high	*Control:* high	*Control:* low	*Control:* high	*Control:* high	
	*Control:* low								
Bias in selection of the reported result	Low	Low	Low	Low	Low	Low	Some concerns	Some concerns	
Overall risk of bias judgement	High	Low	High	High	High	Low	High	High	

Abbreviations: AOFAS, American Orthopaedic Foot and Ankle Society; ASC, adipose‐derived stem cell; ASES, American Shoulder and Elbow Surgeons; BMAC, bone marrow aspirate concentrate; BM‐MSC, bone marrow‐derived mesenchymal stem cell; CMS, Constant–Murley Score; EQ‐5D, EuroQol‐5D; MRI, magnetic resonance imaging; MSC, mesenchymal stem cell; PRP, platelet‐rich plasma; ROM, range of motion; SANE, single assessment numeric evaluation; SF‐12, 12‐item short form health survey; SF‐36, 36‐item short form health survey; US, ultrasound; VAS, visual analogue scale; VISA‐A, Victorian Institute of Sport Assessment – Achilles; VISA‐P, Victorian Institute of Sport Assessment – Pain; VR‐12, veterans RAND 12‐item health survey.

## DISCUSSION

In this review of randomised trials of therapeutic MSC interventions for tendon pathology we found a particularly poor consideration of post‐MSC injection rehabilitative frameworks and descriptions of tissue loading protocols/processes. This was highlighted by a mean TIDieR reporting score of only 2.38 of a possible 12 across the eight RCTs that were eligible for inclusion. Of these studies, the maximum score seen in any single trial was 6/12, while a third of trials scored 0/12.

The eight RCTs reported various MSC preparations that were applied to various body regions, but all trials were designed to evaluate the efficacy and clinical outcomes of the biologic preparations studied, with outcome measures reported out to a minimum of 6‐months post intervention (and up to 2‐years in some trials). The very low TIDieR scores described here, suggest that either rehabilitation parameters, and the mechanotransduction principles that underpin tendon repair, were not considered as a part of the trial MSC intervention, or, that they were considered to be part of the intervention, but reported especially poorly in the trial publications. Either conclusion is problematic. The study designs all contrasted an MSC preparation against placebo or another agent, such as PRP or corticosteroid; Physical rehabilitation was not identified as a control or comparator arm in any trial. No trials discuss the rehabilitation intervention in their reports nor make the link between tendon loading and clinical outcomes through mechanotransduction.

The TIDieR checklist was created to address poor intervention documentation in clinical trials [11]. Unfortunately, despite progress, our finding of incomplete intervention detail is commonplace and widespread. For example, Webster et al. highlight that eight of 12 TIDieR items are adequately reported in placebo and sham‐controlled trials in the leading specialist medical journals [36]. An average score of 8/12 in TIDieR domain reporting is typically reported in the rehabilitation trial literature [4, 17, 18], where the reporting of sufficient detail that would enable proper replication of studies incorporating an exercise intervention remains a problem. In our review the standard deviation, unusually, exceeds the mean value of summed reported items. A standard deviation of 2–3 point/items is typical across the wider literature reflecting typical individual trial TIDieR scores being in the 6–11 range. Our standard deviation range is 0–5 items, further highlighting the particularly poor intervention descriptions in this field.

Whether the trials included in this review considered tissue loading as a part of the intervention or not, none of the protocols offered can be meaningfully reproduced. Only three of the included trials [8, 24, 25] offered some detail as to what rehabilitation was actually carried out, providing a narrative outline that describe differing phases of rehabilitative intervention, based on numbers of weeks post‐intervention. Notably the TIDieR domains relating to intervention tailoring, modifications or reporting of intervention fidelity were not reported by any study, perhaps highlighting again the absolute focus on the biologic as the trial intervention with scant consideration as to how the therapeutic would be integrated into tissue repair beyond the primary injection/seeding.

Both tendon tissue and MSC differentiation are dependent on mechanical stimulus [21, 35]. This mechanotransductive nature is the basis for wider tendon rehabilitation, as exercise protocols attempt to direct the amount of mechanical stimulus provided to the injured area [10]. By disregarding the effect of load on both the target tissue and the MSC intervention, an argument can be made that none of the included studies provided an appropriate environment for their intervention to succeed. This is exacerbated by the lack of discussion surrounding the potential treatment effect a structured rehabilitation protocol may have on their outcomes. By failing to control for load exposure and denying the tendon tissue consistent mechanical stimulus, the studies may be doing their interventions a substantial disservice when evaluating effectiveness. A lack of loading may well negatively impact the clinical outcomes. This is particularly relevant as there is also discrepancy as to the stated efficacy across the included trials, with many studies reporting no benefit of their MSC intervention. Ultimately it is very difficult to draw any conclusions as to MSC efficacy in tendon healing based on the reporting and methodological quality of trials that have been carried out.

### Limitations

We acknowledge that, although we have performed a thorough search of the four major databases where trials of MSC therapies for tendon injury are most likely to be found, our included studies may not be an exhaustive list. We also acknowledge that there is some debate as to whether the TIDieR checklist is the ‘best’ framework for evaluating the completeness of intervention reporting for trial rehabilitation, however this is the tool recommended by the Equator network and is well‐established in the rehabilitation literature base. In terms of the purpose of this review it is an appropriate tool to quantify the extent to which the loading parameters were incorporated in the trial interventions.

## CONCLUSIONS

Both tendons and MSCs require mechanical stimulus for regeneration and repair, however randomised trials of MSCs for tendon pathologies seemingly do not consider this properly in their trial designs. As such, it is debatable to what extent the interventions in the studies have truly been assessed, and efficacy remains unknown. Better trials are urgently needed to determine the effectiveness of MSC interventions.

## AUTHOR CONTRIBUTIONS

Ben Dyck, Gordon J. Hendry and David F. Hamilton contributed to the conception and design of the review. Ben Dyck performed the initial search of databases. Ben Dyck and David F. Hamilton screened and selected the eligible studies. Ben Dyck, Graeme P. Hopper and Chris Clifford performed the data extraction, which was verified by David F. Hamilton. Ben Dyck and David F. Hamilton assessed the risk of bias of the included studies. Ben Dyck and David F. Hamilton drafted the manuscript. All authors reviewed, provided critical revisions, refined and approved the final manuscript.

## CONFLICT OF INTEREST STATEMENT

The authors declare no conflicts of interest.

## ETHICS STATEMENT

As a systematic review of published data this work required no ethical approval.

## Supporting information

Supporting information.

## Data Availability

The data and materials necessary to reproduce the findings reported in this article are available at https://osf.io/b6ptu.
