# Supplementary material for: Poor consideration of tissue loading in randomised trials of MSC interventions for tendon pathology: A systematic review using the TIDieR framework
Source: J Exp Orthop. 2025 Jul 27;12(3):e70388. doi: 10.1002/jeo2.70388 (PMC12296693; doi:10.1002/jeo2.70388)
Supplement: Supplementary file 1 — Supporting information. [file JEO2-12-e70388-s001.docx]

**SUPPLEMENTAL MATERIAL**

**Study PICO**

Population: human tendon injuries

Intervention: rehabilitation following Mesenchymal Stem Cell (MSC) interventions

Comparator: any (alternate intervention, placebo, none or usual care)

Outcome: tendon healing

**Database searches**

| Database: CINAHL Plus with Full Text & MEDLINE | Search Phrase  *MH = Exact subject heading* |
| --- | --- |
| S1 | (MH "Stem Cells") OR "stem cells" OR (MH "Mesenchymal Stem Cells") OR (MH "Adult Stem Cells") OR (MH "Induced Pluripotent Stem Cells") OR (MH "Pluripotent Stem Cells") OR (MH "Multipotent Stem Cells") |
| S2 | (MH "Wounds and Injuries") OR (MH "Athletic Injuries") OR (MH "Ankle Injuries") OR (MH "Elbow Injuries") OR (MH "Shoulder Injuries") OR "injury" OR (MH "Wrist Injuries") OR (MH "Knee Injuries") OR (MH "Leg Injuries") OR (MH "Forearm Injuries") OR (MH "Arm Injuries") OR (MH "Hand Injuries") OR (MH "Foot Injuries") OR (MH "Rotator Cuff") OR "rotator cuff" OR (MH "Rotator Cuff Injuries") OR (MH "Shoulder Impingement Syndrome") OR (MH "Rupture") OR "rupture" OR "lesion" OR "tear" |
| S3 | (MH "Tendons") OR (MH "Hamstring Tendons") OR (MH "Achilles Tendon") OR (MH "Patellar Ligament") OR "tendon*" OR (MH "Tendon Injuries") OR (MH "Tenotomy") OR (MH "Tennis Elbow") OR (MH "Elbow Tendinopathy") OR (MH "Tendinopathy") OR "tendinopathy" |
| S4 | "random*" OR (MH "Randomized Controlled Trials as Topic") |
| S5 | S1 AND S2 AND S3 AND S4 |

| Database: SPORTDiscus With Full Text | Search Phrase  *DE = Descriptors (subjects)* |
| --- | --- |
| S1 | (DE "HUMAN stem cells" OR DE "BONE marrow cells" OR DE "FAT cells") AND (DE "ACHILLES tendon" OR DE "ACHILLES tendon rupture" OR DE "ACHILLES tendon injuries" OR DE "ACHILLES tendinitis" OR DE "TENDON surgery" OR DE "TENDON injuries" OR DE "SOFT tissue injuries" OR DE "PATELLAR tendon" OR DE "PERONEAL tendons" OR DE "TENOSYNOVITIS" OR DE "TENDONS" OR DE "TENDINOPATHY" OR DE "TENDINOSIS" OR DE "JUMPER'S knee" OR DE "DE Quervain disease" OR DE "ACHILLES tendinitis" OR DE "TENNIS elbow" OR DE "GOLF elbow" OR DE "ROTATOR cuff" OR DE "ROTATOR cuff injuries" OR DE "ROTATOR cuff -- Diseases" OR DE "SHOULDER joint" OR DE "SHOULDER injuries" OR DE "SHOULDER joint injuries" OR DE "ELBOW injuries" OR DE "KNEE injuries" OR DE "KNEE diseases" OR DE "KNEE injury treatment" OR DE "WRIST injuries" OR DE "HIP joint injuries" OR DE "HIP joint") |
